# The need for biochemical testing in beta‐enolase deficiency in the genomic era

**DOI:** 10.1002/jmd2.12070

**Published:** 2019-09-03

**Authors:** Ralph Wigley, Renata S. Scalco, Alice R. Gardiner, Richard Godfrey, Suzanne Booth, Richard Kirk, David Hilton‐Jones, Henry Houlden, Simon Heales, Ros Quinlivan

**Affiliations:** ^1^ Enzyme Laboratory, Department of Chemical Pathology, Cameilia Botnar Laboratories Great Ormond Street Hospital for Sick Children London UK; ^2^ MRC Centre for Neuromuscular Diseases and Department of Molecular Neuroscience University College London Institute of Neurology and National Hospital for Neurology and Neurosurgery London UK; ^3^ CAPES Foundation Ministry of Education Brazil; ^4^ Centre for Human Performance, Exercise and Rehabilitation Brunel University London Uxbridge UK; ^5^ Sheffield Diagnostic Genetic Service Sheffield Children's NHS Foundation Trust Sheffield UK; ^6^ Department of Clinical Neurology West Wing, John Radcliffe Hospital Oxford UK; ^7^ Dubowitz Neuromuscular Centre Great Ormond Street Hospital for Children, NHS Foundation Trust London UK

**Keywords:** *ENO3*, glycogen storage disease type XIII, kinetic profile, β‐enolase deficiency

## Abstract

Glycogen storage disease type XIII (GSDXIII) is a very rare inherited metabolic myopathy characterized by autosomal‐recessive mutations in the *ENO3* gene resulting in muscle β‐enolase deficiency, an enzymatic defect of the distal part of glycolysis. Enzyme kinetic studies of two patients presenting with exertion intolerance and recurrent rhabdomyolysis are reported. Next generation sequencing confirmed patient 1 was homozygous for p.E187K in *ENO3*, while patient 2 was homozygous for p.C357Y. *ENO3* variants pathogenicity was confirmed by functional studies in skeletal muscle. p.E187K caused extremely low total enolase activity. p.C357Y was associated with a higher level of residual activity but kinetic studies showed a lower maximum work rate (*V*_max_). This study illustrates that GSDXIII may be caused by either null mutations leading to β‐enolase deficiency or by mutations that alter the enzyme's kinetic profile. This study highlights the importance of carrying out functional studies as part of the diagnostic process following the identification of variants with next generation sequencing.

**Abbreviations**
CK: creatine kinase
GSDXIII: glycogen storage disease type XIII
P: patient
RM: rhabdomyolysis

## 1 INTRODUCTION

Glycogen storage disease type XIII (GSDXIII) is a very rare inherited metabolic myopathy characterized by an enzymatic defect of the distal part of glycolysis; to date only four patients have been reported in the literature^1^, ^2^ The condition is caused by recessive mutations in *ENO3* (OMIM # 131370) resulting in muscle β‐enolase deficiency. Symptoms include exercise intolerance with myalgia as the predominant symptom and recurrent rhabdomyolysis (RM). Symptoms are generally milder than McArdle disease, the most common glycogen storage disorder affecting skeletal muscle. Diagnosis of GSDXIII is by muscle enzymology and molecular genetics.

Here we report the results of enzyme analysis of β‐enolase activity in skeletal muscle tissue from two patients with *ENO3* mutations. The individuals reported symptoms of exercise intolerance, recurrent RM and normal serum creatine kinase (CK) levels in between episodes of RM.

## 2 METHODS

Patients were identified as carrying *ENO3* mutations following genetic analysis on a next generation sequencing panel of genes for acute RM.^3^

All muscle samples were homogenized (glass: glass 0.1 mm clearance) in ice‐cold 0.1 M K_2_HPO_4_ pH 8.0 buffer and centrifuged at 13000 rpm for 3 minutes in a fixed angle rotor. The supernatant was retained and split in two aliquots, the first diluted 2‐fold with ice‐cold 0.1 M K_2_HPO_4_ pH 8.0 buffer for protein analysis by the bichronic acid method with Bovine serum albumin as the standard.^4^ The second aliquot was 2‐fold diluted with ice‐cold 0.1 M K_2_HPO_4_ pH 8.0 buffer with 4 mM dithiothertol for enzyme analysis.

Total enolase activity was measured using a linked enzyme assay monitoring the decrease in fluorescence due to the conversion of NADH to NAD^+^. The assay buffer was at pH 7.4 containing Imidazole (125 mM), NADH (750 μM), KCL (400 mM), and MgSO_4_ (3 mM). ADP was added as a cofactor for pyruvate kinase (6 mM). Pyruvate kinase and lactate dehydrogenase were added as auxiliary enzymes at final activities of 14.3 U/L, and 27.5 U/L. Reactions were carried out at 30°C and initiated by addition of 2‐phosphoglycerate to a final concentration 3.8 mM.

For analysis of kinetic parameters a control pool of five unaffected skeletal muscle homogenates was prepared by the method above and both the control pool and patient samples diluted to a final protein concentration of 0.5 mg/mL. Enzyme activity measurements were made using the above method however the 2‐phosphoglycerate concentrations ranged between 0.19 and 3.8 mM. Results were plotted and fitted to the Michaelis Menten equation using Graph Pad Prism, Version 7.

## 3 RESULTS

### 3.1 Case series

Patient one (P1) was previously reported by Masumeci et al,^1^ and is a 42‐year‐old Turkish male with consanguineous parents, who presented with exercise related muscle pain and RM following sporting activities such as football during childhood (highest documented serum CK level: 75000 IU/L), but with normal baseline serum CK (105 IU/L; reference range: 38‐204 IU/L). An ischemic forearm exercise test, performed many years before his diagnosis was inconclusive with a suboptimal rise in lactate with no rise in ammonia. Muscle biopsy was reported as showing normal histology but a mild increase in glycogen was seen on electron microscopy. Genetic studies confirmed him to be homozygous for a c.559G>A p.E187K, resulting in the glutamate residue 187 being replaced with a lysine (p.E187K). Patient two (P2) is a 23‐year‐old Asian male who presented with recurrent RM (highest documented CK: 193000 IU/L). The first RM episode occurred at 14 years of age. He had normal baseline serum CK (151 IU/L; reference range: 38‐204). Muscle biopsy showed minimal nonspecific changes. A nonischemic forearm exercise test revealed a maximum lactate increase of 3.81 mmol/L. Genetic studies confirmed that he was homozygous for c.1070G>A mutation in exon 10 resulting in p.C357Y substation of the cysteine at position 357 with a tyrosine. In silico modeling using SIFT, PolyPhen2, SNPS 3D, SNPS&GO, PROVEAN predicted the first mutation pE187K to be deleterious as it is highly conserved residue. Insilco analysis for the C357Y mutation using the same software was inconclusive and, therefore, assigned as a variant of unknown significance (VUS).

VUS presents a major diagnostic challenge in the genomic era. Whole exome panels can lead to findings of multiple VUS, often the clinician phenotype could be attributed to more than one VUS. As powerful as the predictive software is for characterizing VUS, the only accurate way to assign pathogenicity is through functional studies. These can include measurement of substrate accumulation, of similar byproduct build up, histological staining, or direct measurement of enzyme activity. Even when in silico model predicts a mutation to be deleterious if that mutation has not been previously reported (as was the case with the E187K mutation), it is advised able to confirm this prediction using functional studies.

### 3.2 Biochemical studies

#### 3.2.1 Muscle enolase activity

**Figure 1:**
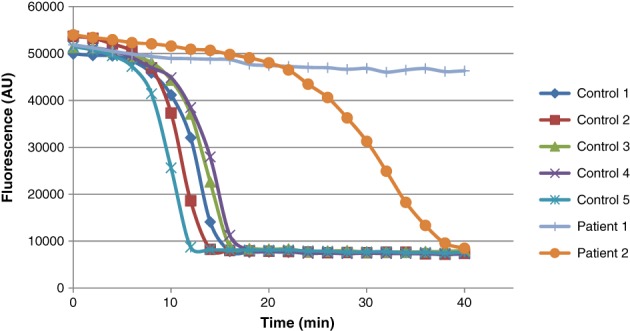
Reaction profile for total enolase activity. Expressed as fluorescence change vs time. Patient 1 (short vertical lines) shows little activity, patient 2 (circles) shows a slow decrease in fluorescence with a marked lag in the start of the reaction compared to the normal controls

Biochemical studies performed on skeletal muscle tissue showed severely diminished total enolase activity in both patients (Table 1). The muscle tissue from P2 had higher residual activity, and the profile of the fluorescence change against time for the reaction was abnormal when compared with simultaneous normal controls (Figure 1). There was a significant lag in the time for the reaction to initiate: 18 minutes in the affected tissue compared to 9 minutes in the controls. Following initiation, the rate of reaction was also slower than the controls.

**Table 1:** Total enolase activity of five controls and patients 1 and 2

Muscle biopsy	Enolase activity (μmol/min/mg ptn)	Phosphoglucomutase activity (μmol/min/mg ptn)
Control 1	286	421
Control 2	316	364
Control 3	305	345
Control 4	270	424
Control 5	382	655
Patient 1	8	263
Patient 2	98	386

*Note*: Patient 1 has <3% the activity of the normal controls while patient 2 has ~33% activity of the normal controls. Phosphoglucomutase activity was measured as a control of sample integrity (normal reference interval > 150 μmol/min/mg ptn).

Abbreviation: ptn, protein.

#### 3.2.2 Kinetic studies

**Figure 2:**
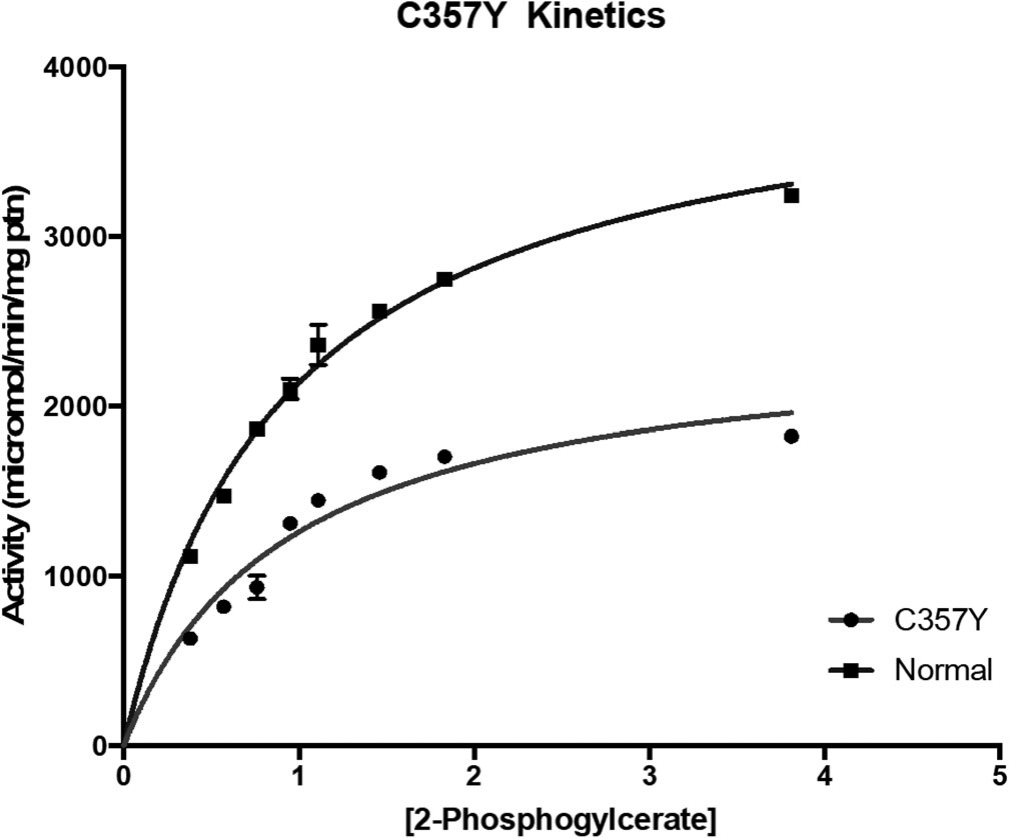
Muscle enolase kinetics for patient 2, and a pool of five normal samples. Data fitted to the Michaelis Menten equation using Graph Pad Prism, Version 7

As there was significant residual enzyme activity in patient two, we decided to investigate further to elucidate if this mutation is pathogenic or not by looking at the kinetic characteristic of the enzyme in the muscle homogenate compared to unaffected samples. The homogenate preparations of muscle tissue from P2 and the pooled normal homogenates had kinetic profiles that had a good fit with the Michaelis Menten equation (see Figure 2). The Michaelis parameter given in Table 2 indicate that the *K*_m_ for 2‐phosphoglycerate remains unchanged by the p.C357Y mutation, however the *V*_max_ of the enzyme‐catalyzed reaction was half that of the pooled normal samples.

**Table 2:** Kinetic parameters of patient 2 and pool of five normal controls

Sample	*K*_m_ (μmol/L)	*V*_max_ (μmol/min/mg ptn)
Pool of normal controls (N = 5)	0.92	4113
C357Y	0.94	2445

*Note: K*_m_ in patient 2 is comparable to normal controls, meaning there is no change in the affinity of the total enolase in patient 2 for 2‐phosphoglycerate. The *V*_max_ in patient 2, however is ~50% that of the normal controls.

## 4 DISCUSSION

GSDXIII is a rare cause of recurrent RM. Here we report our findings on a previously confirmed patient as well as a new patient with recurrent symptoms who was detected by next generation sequencing. Baseline CK levels in both patients were normal but much raised during episodes of RM. Forearm exercise testing revealed a blunt rise in lactate. Lactate increase was higher in P2. P2 responses were tested through a nonischemic forearm test, as this has superseded the use of ischemic forearm exercise testing.^5^ The identified mutations were previously unreported, and in both cases confirmation of diagnosis allowed the clinical team to appropriate advice patients on activity to prevent episodes of RM and improve aerobic capacity.

**Figure 3:**
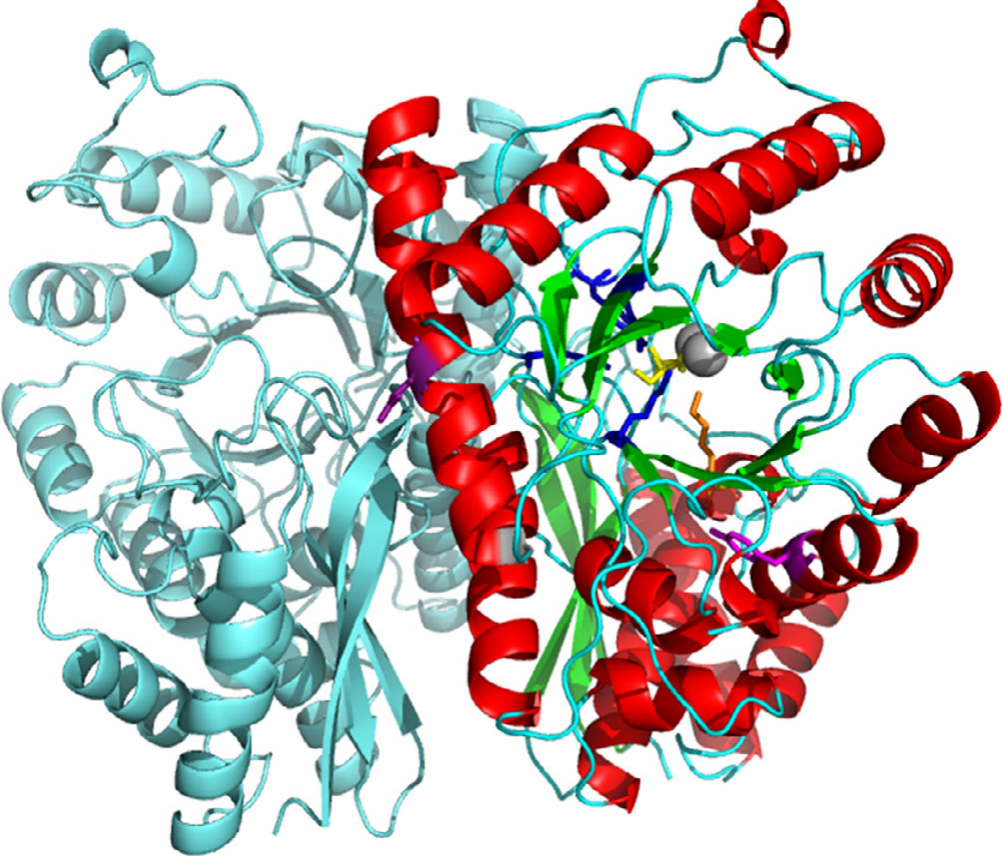
Homodimer of enolase, one dimer shown as a cyan ribbon, second dimer colored showing secondary structure, residue Glu187 shown in purple. Magnesium ions shown as grey spheres with 2‐phosphoglycerate represented as yellow sticks. The side changes of substrate binding residues His 156, Glu 167, Glu 210, Lys 394 shown as blue sticks, catalytic residue side chain Lys 343 represented by orange sticks. Cystine 357 has been mutated to tyrosine the side chain resented by pink sticks. Image generated using the PyMOL Molecular Graphics System, Version 1.3 Schrödinger, LLC, PDB code 3UCC

Biochemical studies on muscle tissue from both patients' confirmed diminished total enolase activity. Total enolase activity in P1 was virtually absent (≤10% residual activity) as previously reported in Musumeci et al.^1^ Glutamate 187 is located on the dimer interface in enolase (Figure 3) and hydrogen bonds with back bone nitrogen of tyrosine 57 of the other chain. This interaction along with hydrogen bond formed by the neighboring residue arginine 183 perhaps stabilizes the dimer:dimer interface. The mutation may disrupt the formation of the hydrogen bonds resulting in the dimer not being formed, and if the monomeric form of enolase is less stable it would be quickly degraded accounting for the low activity observed.

From the reaction profile and the kinetic parameters obtained from analysis of the biopsy from P2, it is evident that the reported mutation did not result in complete loss of enzyme activity. Instead, the mutation resulted in a slower rate of beta‐enolase activity. Plasma lactate raise assessed by the nonischemic forearm exercise test further support the finding of a functional enzyme in skeletal muscle. We hypothesize that the mutation alters the active site of the enzyme, by moving the catalytic residue from its ideal position. While leaving the three‐dimensional confirmation of the residues responsible for the binding of the 2‐phosphogylcerate substrate, intact, this would account for the lowered *V*_max_ with an unaltered *K*_m_ (see Figure 2). One suggestion would be that this 50% decrease in *V*_max_ would not cause any muscle symptoms during low energy demand; however when initiating exercise or during very intense (anaerobic) exercise, there may be inhibition of the maximal rate of enzyme activity resulting in a “bottle neck” in the pathway leading to the observed symptoms.

## 5 CONCLUSION

Mutations in *ENO3* can result in either low total enolase activity in skeletal muscle tissue (≤10%) or higher residual activity but with an altered kinetic parameter of the enzyme resulting in either reduced availability or working capacity of the enzyme for substrate metabolism. This study illustrates the importance of using functional studies to confirm novel mutations identified on next generation sequencing panels. As with all diagnostic tools, functional studies should be used to answer the clinical question as best as possible. This is likely to be as part of a multidisciplinary team approach in complex cases such as theses.

## CONFLICT OF INTEREST

Ralph Wigley, Renata S. Scalco, Alice R. Gardiner, Richard Godfrey, Suzanne Booth, Richard Kirk, David Hilton‐Jones, Henry Houlden, Simon Heales, and Ros Quinlivan declare that they have no conflict of interest.
